# Display of receptor-binding domain of SARS-CoV-2 Spike protein variants on the *Saccharomyces cerevisiae* cell surface

**DOI:** 10.3389/fimmu.2022.935573

**Published:** 2022-08-12

**Authors:** Hongguan Xing, Liyan Zhu, Pingping Wang, Guoping Zhao, Zhihua Zhou, Yi Yang, Hong Zou, Xing Yan

**Affiliations:** ^1^ Shanghai Key Laboratory of Chemical Biology, School of Pharmacy, East China University of Science and Technology, Shanghai, China; ^2^ Chinese Academy of Science-Key Laboratory of Synthetic Biology, Chinese Academy of Science Center for Excellence in Molecular Plant Sciences, Institute of Plant Physiology and Ecology, Chinese Academy of Sciences, Shanghai, China; ^3^ Key Laboratory of Medical Molecular Virology of Ministry of Education/National Health Commission, School of Basic Medical Sciences, Shanghai Medical College, Fudan University, Shanghai, China; ^4^ Department of Process Technology, Zhejiang Hongguan Bio-pharma Co., Ltd., Jiaxing, China; ^5^ Chinese Academy of Sciences Engineering Laboratory for Nutrition, Shanghai Institute of Nutrition and Health, Chinese Academy of Sciences, Shanghai, China

**Keywords:** SARS-CoV-2, receptor-binding domain, yeast surface display, vaccine, B.1.671.1

## Abstract

Severe acute respiratory syndrome coronavirus-2 (SARS-CoV-2), the causative agent of coronavirus disease 2019 (COVID-19), represents a significant global human health threat. The most effective way to end the pandemic is through timely vaccination. In this study, the receptor-binding domains (RBDs) of Spike protein of the initial strain of SARS-CoV-2 and its variants, B.1.1.7 (Alpha), B.1.351 (Beta), and B.1.617.1 (Kappa), were successfully displayed on the surface of a *Saccharomyces cerevisiae* strain for development as a vaccine candidate. To rapidly express the recombinant protein and avoid the need for expensive galactose as an inducer, the *gal80* gene of *S*. *cerevisiae* was knocked out, and the conventional 72-h culture period was thus successfully shortened to 24 h. Mice vaccinated against variant B.1.617.1 showed robust humoral and cellular immune responses. Moreover, the antiserum in the B.1.671.1 group had neutralizing activity against wild-type RBD and high binding titers against RBD mutants of variants B.1.351 and B.1.1.7. Double deglycosylation at N331Q and N343Q resulted in marked reduction of the affinity of RBD binding to angiotensin converting enzyme 2 (ACE2) and escaped antibody neutralization. This study demonstrates that yeast surface display technology can provide an alternative approach to rapid large-scale preparation of promising SARS-CoV-2 vaccine candidates at low cost.

## Introduction

Severe acute respiratory syndrome coronavirus-2 (SARS-CoV-2), the causative agent of coronavirus disease 2019 (COVID-19), has caused a global pandemic that, up to early July 2022, has infected more than 544 million people and has caused more than 6.3 million deaths around the world. This virus poses a serious global public health emergency. Developing vaccines against COVID-19 is considered the most promising approach for curbing the pandemic. Many teams around the world have already been engaged in SARS-CoV-2 vaccine development (1, 2). Several vaccines have been shown to be effective and have been approved for use in different countries (3). Most of these were developed on one of five different platforms: protein/peptide vaccines, nucleic acid vaccines (mRNA/DNA), viral vector vaccines, virus-like particle vaccines, and live-attenuated/inactivated vaccines. Each of these platforms has both advantages and disadvantages in terms of production, scaling up, and safety (4). For example, the preparation of mRNA vaccines is simple, but scaling up is both difficult and costly (5). The instability of the mRNA in serum and lack of effective mRNA delivery systems increase production costs (6). In addition, the “cold chain” storage of vaccines is also a major factor restricting effort to scale up production; e.g., COVID-19 mRNA vaccine candidates from Pfizer and BioNTech must be stored at –70°C during the entire distribution process from the manufacturer’s stores to the intended destination (7). Recently, the AstraZeneca DNA vaccine based on an adenovirus vector was reported to cause thromboembolic events (8). By the end of 2021, the scale of the global vaccine market was estimated to be around 12 billion doses, while only about 413 million doses had been produced by the beginning of March (5). More importantly, SARS-CoV-2 is a single-stranded RNA virus that easily accrues mutations during transmission. Several potentially more dangerous SARS-CoV-2 variants have been identified and are now circulating worldwide, including the B.1.1.7 (Alpha) lineage first identified in the UK (9), the B.1.351 (Beta) lineage first identified in South Africa (10), and the B.1.617 (Delta) lineage first identified in India (11). These variants, especially B.1.617, have shown marked reduction of neutralization by the vaccine sera (12, 13), indicating that SARS-CoV-2 is mutating faster than vaccines can be developed. Therefore, the development of a convenient, fast, and inexpensive vaccine production platform would be valuable.

*Saccharomyces cerevisiae* is the best-understood eukaryote at the molecular level, and technologies for its large-scale fermentation are well established (14, 15). Yeast-based expression systems have both the advantages of prokaryotes, such as high expression levels, ease of scaling up, and low cost, and the advantages of eukaryotes in performing posttranslational modifications (16). In addition, *S. cerevisiae* is a Generally Recognized as Safe organism, which is not affected by the endotoxin problem of bacterial expression systems and risk of virus contamination associated with mammalian expression systems. Furthermore, the cellular components of *S. cerevisiae*, such as the polysaccharide β-1,3-d-glucan (BG) and mannan, can be used as natural adjuvants with the ability to stimulate or modulate the immune response (17). With regard to storage and distribution, yeast also has advantages for long-term preservation of vaccines under non-refrigerated conditions (18). Therefore, *S. cerevisiae* is considered a promising delivery platform for the development of human and veterinary vaccines. Many studies have yielded encouraging results with yeast-based vaccines against common viruses, such as hepatitis B virus (19), H7N9 virus (20), porcine epidemic diarrhea virus (21), and SARS-CoV-2 (22).

Here, we report the display of SARS-CoV-2 recombinant protein antigen and variants on the cell surface of *S*. *cerevisiae* and describe its characterization as a potential vaccine. RBDs (R318-N531) of wild-type (Wuhan-Hu-1 strain) SARS-CoV-2 Spike protein and the B.1.1.7, B.1.351, and B.1.671.1 variant mutant RBDs (mRBDs) were fused to the C-terminus of the *S*. *cerevisiae* Aga2 protein (one of the subunits of the **a**-agglutinin receptor). The *gal80* gene of the yeast strain EBY100 was knocked out to achieve rapid expression of the recombinant protein using glucose as a carbon source instead of the more expensive galactose. Western blotting analysis and immunofluorescence microscopy confirmed the display of RBD fusion protein on the *S*. *cerevisiae* surface after 24 h in culture. Immunization experiments in mice indicated that variant B.1.671.1 triggered host humoral and cellular immune responses. In addition, by construction of deglycosylated mimetic N331Q and N343Q variants of wild-type RBD, we confirmed that glycosylation of the RBD is essential for its binding affinity to the receptor, ACE2.

## Materials and methods

### Plasmids, strains, and media

pYD1 plasmid (Invitrogen, San Diego, CA) was used to construct SARS-CoV-2 RBD surface display vector. *Escherichia coli* Top10 was the host strain used for the construction of expression vector and propagated in Luria–Bertani (LB) broth (1% peptone, 0.5% yeast extract and 1% sodium chloride) at 37°C. *S. cerevisiae* EBY100 (Invitrogen, San Diego, CA) served as a host cell for RBD and its variants’ surface display and grown in YNB-CAA-Glucose medium (0.67% yeast nitrogen base without amino acids, 0.5 casamino acids, 2% glucose) at 30°C. Minimal dextrose medium (0.67% yeast nitrogen base without amino acids, 2% dextrose, 0.01% leucine, 2% agar) was used to select yeast transformants.

### Construction of surface display plasmids and expression of proteins

The SARS-CoV-2 Spike RBD (S protein 318-531, GenBank: YP_009724390) encoding gene was synthesized by GenScript (Nanjing, Jiangsu, China) and subcloned into *Bam*HI endonuclease sites of pYD1 plasmid. The variants B.1.1.7 (N501Y), B.1.351 (N501Y/E484K/K417N), and B.1.617.1 (L452R/E484Q) were constructed by a site-directed mutagenesis kit (see Section 2.7) with temple of wild-type RBD. The *S. cerevisiae* EBY200 strain was constructed by deleting the gal80 gene of *S. cerevisiae* EBY100 strain. The resultant shuttle plasmid pYD1-RBD was transformed into *S. cerevisiae* EBY200. Recombinant yeast transformants were plated on selective minimal dextrose plates at 30°C for 3 days. The positive clones were confirmed by DNA sequencing and then induced in YNB-CAA-Glucose medium (0.67% YNB, 0.5 casamino acids, 2% glucose) at 30°C for 24 h for RBD expression. Meanwhile, *S. cerevisiae* EBY200 containing empty pYD1 plasmid was used as a negative control for the following tests.

### Vaccine preparation

Two hundred microliters of recombinant *S. cerevisiae* PYD1-RBD/EBY200 and variant cells were harvested after 24 h induction in YNB-CAA-Glucose medium and washed three times in PBS. Collected cells were inactivated at 60°C for 1 h. After treatment, cells were washed three times with PBS and resuspended in 10 ml of sterile 10% skimmed milk and freeze-dried. The freeze-dried cells were stored at 4°C until use. Before immunization, 5 OD 600 nm freeze-dried cell powder was washed three times with 1 ml of PBS and resuspended in 100 μl of PBS.

### Immunofluorescence microscopy

For immunofluorescence assay and microscopy, 1 OD 600 nm of *S. cerevisiae* pYD1-RBD*/*EBY 200 and variant cell pellets were harvested and washed twice with PBS buffer. Remove the PBS and resuspend the cell pellets in 250 µl of PBS, 1 mg/ml BSA, and 1 µg of rabbit anti-RBD (wild-type) antibody (Sino Biological, Beijing, China) on ice for 30 min with occasional mixing. Centrifuge the cells at 5,000 × *g* for 5 min at 4°C and washed twice with PBS. After the cells were washed, the second antibody, fluorescein isothiocyanate (FITC)-conjugated goat anti-rabbit IgG (1 µg; Abcam, UK), was reacted with them on ice for 30 min in the dark and the tube was occasionally inverted. After washing, the cell pellets were resuspended in 40 µl of PBS and spot 10 µl on a slide for immunofluorescence assay (Olympus, Japan). The *S. cerevisiae* EBY200 strain with pYD1 plasmid was used as a negative control.

### Western blot analysis

For Western blot analysis, 1 OD 600 nm of pYD1-RBD/EBY 200 and variants were harvested at 24 h post-induction. The pellets were washed twice with 500 µl of sterile distilled water and re-suspended with 10 µl of 4× SDS loading buffer and 30 µl of sterile distilled water. The suspension was boiled for 15 min. The treated samples were resolved by SDS-polyacrylamide gel electrophoresis and then transferred to nitrocellulose membrane (Sangon, Shanghai, China). Membranes were blocked in TBST buffer (0.1% Tween-20, 150 mM NaCl, and 10 mM Tris, pH 8.3) containing 10% skimmed milk for 1 h at room temperature. After blocking, membrane was incubated with rabbit anti-RBD antibody (Sino Biological, Beijing, China) for 1 h at room temperature followed by three 10-min washes in TBST buffer. The membrane was followed incubated with an HRP-conjugated goat anti-rabbit IgG (YEASEN, Shanghai, China) with 2,500 dilution at room temperature for 1 h. After washing three times with TBST, Tanon High-sig ECL Substrate (Tanon, Shanghai, China) was added to visualize the bands and imaged by Tanon Imager System (Tanon, Shanghai, China).

### Quantification of recombinant protein by indirect enzyme-linked immunosorbent assay

The quantity of RBD expressed in EBY200 surface was determined by quantitative ELISA, as described previously (23). In brief, 0.5 mg of dry power of *S. cerevisiae* pYD1-RBD/EBY200 cells was weighed in 1.5-ml microtubes and washed twice with PBS. Collected cell pellets were resuspended in 100 µl of a series of diluted Anti-Spike-RBD human IgG (Sanyou Bio, Shanghai, China) solutions [in PBS containing 1% (w/v) BSA] and incubated at room temperature for 1 h. Cell pellets were washed three times in PBS containing 0.05% Tween-20. After washing, cells were resuspended in 100 µl of PBS containing 0.1 µg of rabbit anti-human IgG-HRP antibody (Abcam, UK) and incubated at room temperature for 1 h. After washing in the same way, cells were developed with the addition of 100 µl of HRP substrate 3,3′,5,5′-tetramethylbenzidine (TMB) (YEASEN, Shanghai, China) at room temperature in darkness for 30 min and stopped reactions by addition of 100 μl of 2 mol/L H_2_SO_4_. Finally, absorbance of cell supernatant was measured at 450 nm using a microplate reader (Thermo Electron Corporation, USA).

### Site-directed mutagenesis of SARS-CoV-2 Spike RBD

In order to construct RBD deglycosylated expression plasmid, site-directed mutagenesis was performed following the instruction of a site-directed mutagenesis kit (Hieff Mut™, Yeasen, Shanghai, China). Briefly, the plasmid pYD1-RBD was used as the template to generate the mutagenesis in RBD gene of N331 and N343. Following site-directed mutagenesis PCR, the amplification fragments were digested using *Dpn*I at 37°C for 1.5 h. Then, the mixtures were transformed to *E. coli* Top10 for screening.

### Affinity analysis of SARS-CoV-2 Spike RBD and ACE2

To determine the affinity of RBD and its deglycosylated mutants to ACE2, 0.5 OD 600 nm of *S. cerevisiae* pYD1-RBD/EBY200 cell pellets was centrifuged in 1.5-ml microtubes and washed twice with PBS. Subsequently, cells were resuspended in 100 µl of a series of diluted biotinylated Human ACE2 protein (Acro Biosystems, Beijing, China) solutions in PBS containing 1% (w/v) BSA and incubated at room temperature for 1 h. Cell pellets were washed three times in PBS containing 0.05% Tween-20. After washing, cells were resuspended in 100 µl of PBS containing 0.1 µg of Streptavidin-HRP (Acro Biosystems, Beijing, China) and incubated at 37°C for 1 h to avoid light. After washing in the same way, cells were developed with the addition of 100 µl of HRP substrate 3,3′,5,5′-tetramethylbenzidine (TMB) (YEASEN, Shanghai, China) at room temperature in darkness for 30 min and reactions were stopped by the addition of 100 μl of 2 mol/L H_2_SO_4_. Finally, absorbance of cell supernatant was measured at 450 nm using a microplate reader.

### Immunization and sample collection

Female SPF BALB/c mice at 6–7 weeks of age were purchased from Shanghai Laboratory Animal Co. Ltd, China. Mice were housed under a 12:12h light/dark cycle at controlled temperature. Mice were allowed for a week adaptation period upon arrival. All animal experimental protocols were approved by the Animal Ethics Committee of the School of Basic Medical Sciences, Fudan University (approval No. FE21113).

Mice were divided into six groups: PBS control group, vector-only control group (vaccinated with *S. cerevisiae* pYD1/EBY200), inactivation treated surface-displayed RBD (wild) group [vaccinated with inactivated pYD1-RBD (wild)/EBY200], surface-displayed RBD (B.1.351) group [vaccinated with inactivated pYD1-RBD (B.1.351)/EBY200], surface-displayed RBD (B.1.1.7) group [vaccinated with inactivated pYD1-RBD (B.1.1.7)/EBY200], and surface-displayed RBD (B.1.617.1) group [vaccinated with inactivated pYD1-RBD (B.1.617.1)/EBY200]. Each group consisted of six mice. Yeast cells or PBS were injected subcutaneously in the mouse back skin for prime immunization and boosted on day 15, day 30, day 47, and day 69. Blood was collected on day 28, day 44, and day 64 after the initial immunization. Plasma was used for RBD-specific IgG antibody titer detection and neutralization titer detection. Mice were sacrificed 5 weeks after the fifth immunization (D120), and splenocytes were isolated and prepared to detect IFNγ levels (ELISPOT) and multifunctional T-cell response (flow cytometry).

### Antibody titer detection

Binding titer: antigen-specific IgG antibody in plasma was detected by an indirect ELISA as previously described (23). Briefly, microplates (Corning, New York, USA) were coated with 1 μg/ml of wild-type RBD or various mutant RBDs (Sino, Shanghai, China) in 100 µl 0.05 M carbonate-bicarbonate buffer (pH 9.6) and incubated overnight at 4°C. Plates were washed with PBS containing 0.5% Tween 20 (Beyotime, Shanghai, China) and blocked with PBS containing 5% (v/v) fetal bovine serum (FBS) (Hyclone, Utah, USA) at 37°C for 2 h. After washing three times, diluted plasmas (1:50) in PBS containing 0.5% (v/v) FBS were added and incubated for 2 h at 37°C. After three additional washes, 1:5,000 diluted goat anti-mouse IgG horseradish peroxidase conjugate (Proteintech, Chicago, USA) was added and further incubated for 2 h at 37°C. Plates were read at 450 nm on a microplate reader (Thermo scientific, Massachusetts, USA).

### Neutralizing titer detection

Refer to the instructions of the anti-SARS-COV-2 Neutralizing Antibody Titer Serologic Assay Kit (Acrobiosystems, Shanghai, China). Briefly, a 96-well plate was pre-coated with human ACE2 protein and 50 μl of serially diluted sera samples (collected at day 69 and serially diluted twofold starting at a 1:16 dilution); positive control and negative control were added to each well followed by adding 50 μl of HRP-SARS-CoV-2 Spike RBD (wild) working solution (0.3 μg/ml). The plate was sealed with microplate sealing film and incubated at 37°C for 1 h to avoid light. After washing three times, 100 μl of substrate solution was added to each well and the plate was incubated at 37°C for 20 min. Then, 50 μl of stop solution was added to each well. After gently shaking the plate for 3 min, the absorbance at 450 nm was read using a microplate spectrophotometer. Results were judged according to the following criteria: (1) Percent inhibition = (1 − OD 450 of sample/OD 450 of negative control) × 100%; (2) Cutoff value = 20% signal inhibition; (3) Positive: Percent inhibition of sample ≥ Cutoff value; (4) Negative: Percent inhibition of sample < Cutoff value.

### ELISpot assay

The spleen was collected aseptically and was placed in a cell filter (BD Falcon, New York, USA) with a diameter of 70 μM. Then, the spleen was ground with a disposable 1-ml syringe plug and rinsed with RPMI 1640 medium (Hyclone, Utah, USA). Splenocytes were isolated by lymphoprep (Axis Shield, Scotland, UK) according to the instructions.

IFN-γ ELISpot assays were performed using the Ms IFN-Gma ELISPOT Set (BD Pharmingen, Chicago, USA) referring to the manufacturer’s instructions and reference (24). Freshly isolated splenocytes (2 × 10^5^ or 4 × 10^4^) were co-cultured with wild-type RBD (40 µg/ml) or concanavalin A (ConA) (Sigma-Aldrich, Missouri, USA) (5 µg/ml) in 100 µl of RPMI 1640 containing 10% FBS, 1% P/S, 1% HEPES buffer (Gibco, New York, USA), 0.1% 2-mercaptoethanol (Gibco, New York, USA), 1% L-glutamine, and 1% Mem NEAA (Gibco, New York, USA) for 20 h in a humidified 37°C CO_2_ incubator, and because there is no stimuli control, splenocytes were left untreated. Spots were revealed by AEC coloring system (Dakewe, Shenzhen, China) according to the kit manual and determined using an automatic ELISpot reader (CTL, Ohio, USA) and image analysis software (ImmunoSpot 5.1.36). The number of IFN-γ cells was adjusted by subtracting the background number in the no stimuli controls and normalized by the ConA controls based on the following formula: *N*_adjusted_ = (*N*_test_ − *N*_blank_)×*F*, *F* = 2000/[(*N*_ConA_ − *N*_blank_)×5].

### Multifunctional T-cell response

Splenocytes (1.5×10^6^) were inoculated in a 96-well cell culture plate (Corning, New York, USA), co-cultured with 40 μg/ml wild-type RBD for 29 h or positive stimulant PMA (0.1 μg/ml) (Sigma-Aldrich, Missouri, USA) + Ion (1 μg/ml) (Sigma-Aldrich, Missouri, USA) for 6 h. After stimulation, cells were stained referring to the instruction of Cytofix/Cytoperm W/Golgi Stop Kit (BD Pharmingen, Chicago, USA). Briefly, the 96-well plate was centrifugated (2,000 rpm, 5 min) and washed twice with PBS. Then, the cells were blocked on ice in 50 μl of CD16/CD32 antibody (1:100 in 2% FASC Buffer) (BD Pharmingen, Chicago, USA) for 20 min, and stained with fluorescent-labeled anti-CD3, anti-CD4, and anti-CD8 monoclonal antibody (final concentration: 1:400 in 2% FASC Buffer) (BD Pharmingen, Chicago, USA) at 4°C for 40 min. After washing, cells were treated with the fixation and permeabilization solution (4°C, darkness, 20 min). Then, cells were washed twice with PBS, and treated with 50 μl/well fluorescent conjugated IL-2/TNF-α/IFN-γ antibody (final concentration 1:200 in 2% FASC Buffer) (BD Pharmingen, Chicago, USA) at 4°C for 40 min. After washing, cells were suspended with 150 μl of 2% FASC Buffer and analyzed by flow cytometry (Beckman Cytoflex LX, Florida, USA).

## Results

### Expression of RBDs of SARS-CoV-2 Spike protein and its variants on the surface of *S*. *cerevisiae* EBY200

**Figure 1:**
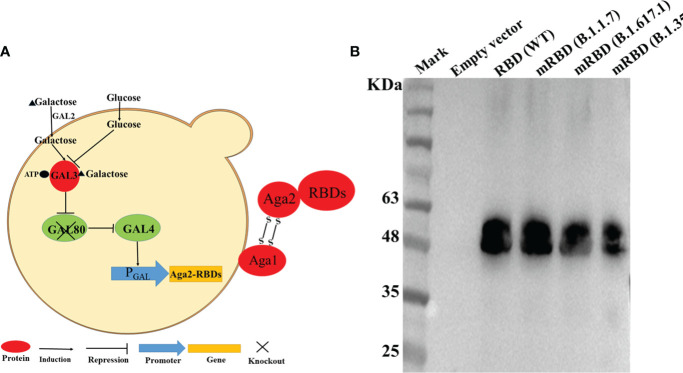
RBD displayed on the *S*. *cerevisiae* surface. **(A)** Schematic illustration of RBD displayed on the yeast surface. **(B)** Western blotting analysis of RBD and its mutant variants expressed on the surface of *S*. *cerevisiae* EBY200.

In this study, we expressed the RBDs of SARS-CoV-2 Spike protein and its mutant variants by engineering *S*. *cerevisiae* EBY100 as a surface-expressing host. The DNA fragments encoding the RBD or mRBDs were cloned into the plasmid pYD1 to express the fusion proteins of RBD and the second subunit of the A-agglutinin receptor (C-terminus of Aga2) (**Figure 1A**). Aga2-RBD and Aga2-mRBD were expected to be expressed under the control of the GAL1 promoter in *S*. *cerevisiae* EBY100, and to bind to Aga1 (another subunit of **a**-agglutinin receptor) through two disulfide bonds while Aga1 was secreted from the yeast cells and covalently attached to β-glucan in the extracellular matrix of the yeast cell wall (**Figure 1A**). Thus, RBD and mRBDs could be displayed on the yeast cell wall (**Figure 1A**). To use glucose instead of galactose as an inducer, the *gal80* gene encoding Gal80 protein, which acts as a negative regulator to repress transcription of the *gal* gene (25) in *S*. *cerevisiae* EBY100, was knocked out, and the resulting strain was designated EBY200. After cultivation in YNB-CAA-Glucose medium for 24 h, the expression of Aga2-RBD fusion protein in EBY200 was analyzed by Western blotting (**Figure 1B**). As expected, a specific band was observed for Aga2-RBD fusion protein (**Figure 1B**), while no band was observed in the negative control (EBY200 strain harboring pYD1 empty vector).

### Immunofluorescence microscopy

**Figure 2:**
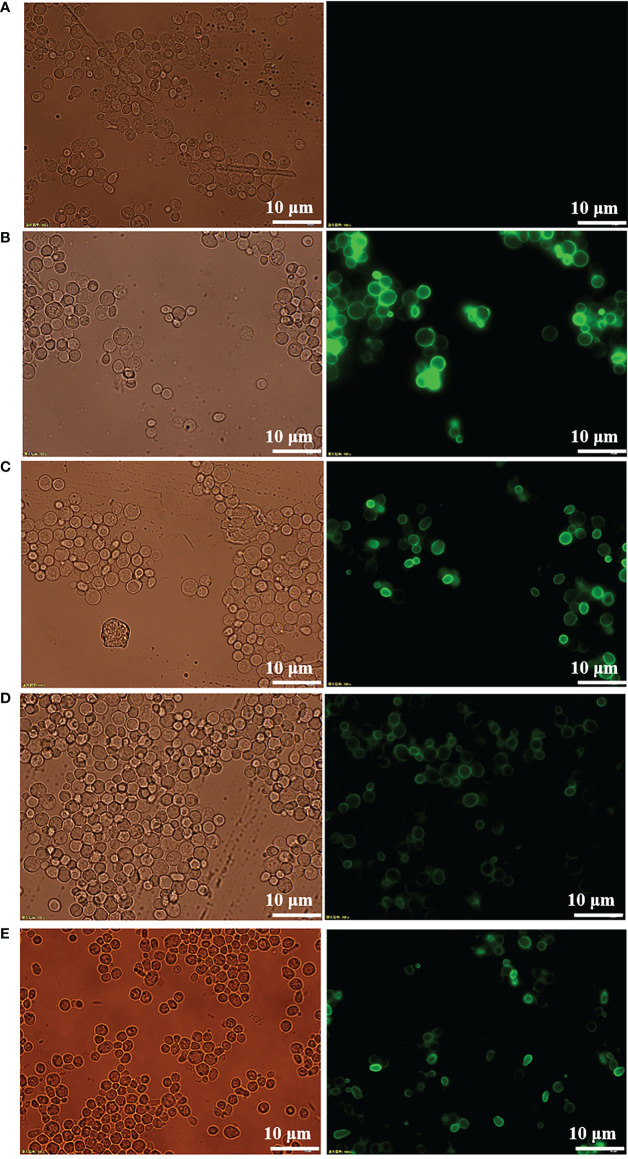
Immunofluorescence microscopy of recombinant yeast cells. **(A)** pYD1/EBY200. **(B)** pYD1-RBD (WT)/EBY200. **(C)** pYD1-mRBD (B.1.17)/EBY200. **(D)** pYD1-mRBD (B.1.617.1)/EBY200. **(E)** pYD1-mRBD (B.1.351)/EBY200 (left: light; right: matching fluorescence, Scale bar: 10 µm).

Expression of recombinant protein in nonnative host organisms can lead to improper secretion and folding. To confirm the proper secretion and correct targeting of RBD and its mutants to the cell surface, immunofluorescence microscopy was performed to visualize the fusion protein. As shown in **Figure 2**, visible green fluorescence was detected in pYD1-RBD/EBY200 and pYD1-mRBD/EBY200 cells after 24 h in culture, but not in negative control pYD1/EBY200 cells grown under the same conditions. The fluorescent signal of the fusion protein appeared as a ring around the cell wall rather than inside the cells, as the antibodies are unable to penetrate into the yeast cell membrane (26). These results show that RBD and its mutants were successfully anchored to the surface of yeast EBY200 cells. In addition, we found that the fluorescence intensity of the fused mRBD of variants B.1.351 and B.1.617.1, especially mRBD of B.1.617.1, was significantly weaker than that of the wild type (**Figures 2D** **, E**). We speculated that this may have been because the antibody used was an anti-wild-type RBD antibody. Both mRBDs from variants B.1.351 and B.1.617.1 have mutations at position E484, which make them more transmissible and also exhibited the greatest reduction in neutralization of the antibody sera induced by wild-type antigen (12, 27). As with the fluorescence intensity results, Western blotting analysis also supported this conclusion (**Figure 1B**). However, the efficacy of the mRBD from variant B.1.17 to neutralizing wild-type antibodies was not reduced, although its transmissibility was higher than that of the wild-type (28). Therefore, the fluorescence intensity of mRBD (B.1.1.7) was consistent with that of the wild type (**Figures 2C**, **1B**).

### Vaccine preparation by heat treatment at 60°C

**Figure 3:**
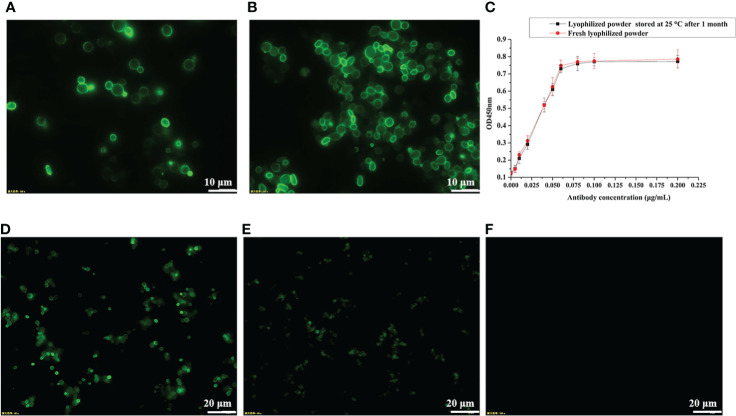
Determination of protein stability by immunofluorescence microscopy and ELISA. **(A)** pYD1-RBD/EBY200 before heat inactivation. **(B)** pYD1-RBD/EBY200 after heat inactivation. **(C)** Determination of protein stability of lyophilized yeast powder at 25°C for 1 month by ELISA. **(D)** Fresh lyophilized yeast powder at 4°C after 3 days. **(E)** Lyophilized yeast power at 4°C after 10 months. **(F)** Fresh lyophilized yeast powder with empty vector pYD1.

For vaccine preparation, we heat-inactivated the recombinant yeast cells at 60°C for 1 h. To confirm whether heat treatment at 60°C would break the disulfide bond between Aga1 and Aga2 (**Figure 1A**) and thus cause the RBD fusion protein to be detached from the yeast cell surface, we measured the fluorescence signal of wild-type RBD recombinant yeast before and after heat inactivation by immunofluorescence microscopy. As shown in **Figures 3A** **, B**, heat inactivation treatment did not affect the anchoring of fusion proteins on the surface of yeast cells. To facilitate vaccine storage, the recombinant yeast cells were lyophilized into a powder, which has been shown to improve stability in routine vaccine preparation (29). The stability of recombinant RBD was determined after 1 month of storage at room temperature by ELISA, and the results indicated that stability of RBD was not compromised (**Figure 3C**). The activity of RBD could be detected by immunofluorescence even after storage at 4°C for more than 10 months (**Figures 3D** **, E**).

### Heat-treated *S*. *cerevisiae* surface-displayed B.1.617.1 mRBD elicits a robust humoral immune response

To investigate whether yeast with surface-displayed SARS-CoV-2 RBD and variants can be used as a vaccine to elicit a protective immune response, mice were immunized subcutaneously with 5 OD inactivated recombinant yeast vaccines in 100 µl of PBS, with the empty vector and PBS serving as negative controls. The RBD-specific IgG levels were measured on days 28, 44, and 64 after the first immunization.

**Figure 4:**
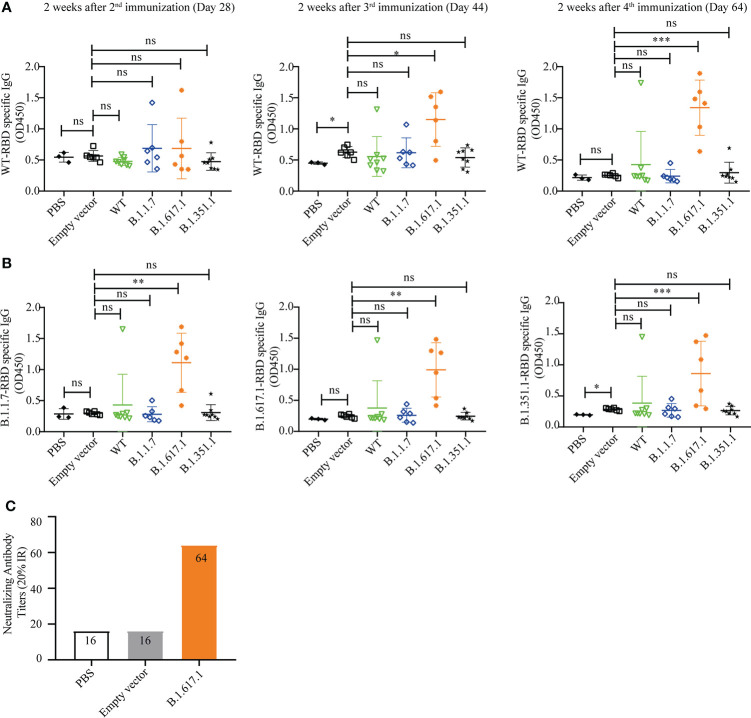
Humoral immune responses elicited by wild-type SARS-CoV-2 Spike RBD and its mutant variants. **(A)** Wild-type RBD-specific IgG serum antibody after 2nd (day 28), 3rd (day 44), and 4th (day 64) immunizations. **(B)** B.1.1.7, B.1.671.1, and B.1.1351 mRBD-specific IgG serum antibody after 4th (day 64) immunization. **(C)** The neutralization titer of B.1.617.1 mRBD antiserum against wild-type RBD was determined by competitive ELISA. Serum samples were collected 2 weeks after the fourth immunization (Day 64), and detected after mixing the samples from the same group in equal volume. Data points represent individual animals. For statistical analysis, *t*-test was performed to compare to PYD1/EBY200 (Empty vector) and PBS groups. Asterisks represent significance: *p < 0.05; **p < 0.01; *n* = 6. ns, no significant; ***: p< 0.001

As shown in **Figure 4A**, there were no significant differences in RBD-specific IgG levels in antisera after second immunization (day 28) in mice vaccinated with wild-type RBD recombinant yeast and three variants compared to the empty vector and PBS groups (*p* > 0.05). However, mice vaccinated with variant B.1.617.1 showed a significant increase in serum RBD-specific IgG level after the third immunization (day 44) and a further increase after the 4th immunization (day 64). Mice vaccinated with wild-type RBD and mRBDs from variants B.1.1.7 and B.1.351.1 showed no significant differences after fourth immunization except for individual mice in which RBD-specific IgG antibodies could be detected.

Subsequently, we coated the mutant RBDs of variants B.1.1.7, B.1.617.1, and B.1.351 to detect the cross-reactivity of antiserum 2 weeks after the fourth immunization (day 64). As shown in **Figure 4B**, sera from mice immunized with mRBDs of variants B.1.1.7 and B.1.351 still did not detect antigen-specific IgG. However, antisera from groups immunized with mRBD of B.1.617.1 were able to bind not only wild-type RBD and B.1.617.1 antigens but also antigens of variants B.1.351 and B.1.1.7.

The neutralization titer of mRBD of B.1.617.1 antiserum against wild-type RBD was determined by competitive ELISA. Serum samples were collected 2 weeks after the fourth immunization (day 64), and detected after mixing the samples from the same group in equal volume. As shown in **Figure 4C**, the sera from groups immunized with mRBD of B.1.617.1 were positive for neutralizing antibodies with a neutralizing titer of 64 (20% IR). By contrast, PBS and empty vector groups were negative. The above results indicate that mRBD of B.1.617.1 vaccine triggers a robust humoral immune response in mice.

### Heat-treated *S*. *cerevisiae* surface-displayed B.1.617.1 mRBD elicits specific T-cell responses

**Figure 5:**
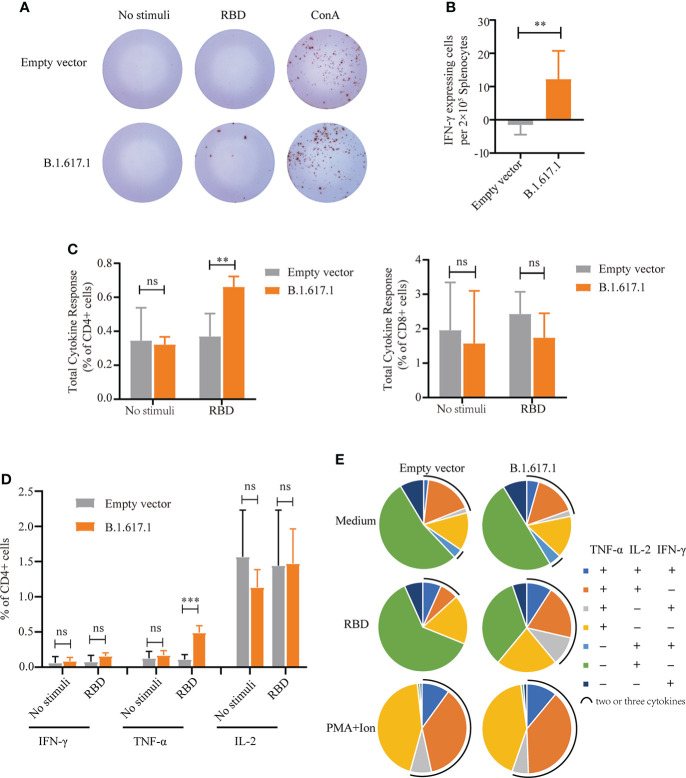
Cellular immune responses in splenocytes from mice vaccinated with yeast surface-displayed B.1.617.1 mRBD. **(A)** The appearance of IFN-γ-expressing spots in an ELISpot assay plate. Each dot represents a single cell secreting IFN-γ. **(B)** Level of IFN-γ expression per 2 × 10^5^ splenocytes. **(C)** The total cytokine response on flow cytometry with intracellular cytokine staining 5 weeks after the fourth immunization or 120 days after priming. The levels of IFN-γ, IL-2, and TNF-α secretion by CD4^+^ and CD8^+^ T cells were quantified for each group and expressed as the frequency of cells expressing any one of the three cytokines. **(D)** Frequency of CD4^+^ T cells positive for each cytokine (IFN-γ, IL-2, and TNF-α) after stimulation with wild-type RBD antigen. **(E)** The proportions of CD4^+^ T cells secreting any combination of IFN-γ, IL-2, and TNF-α after stimulation with wild-type RBD antigen. A *t*-test was performed to compare the mRBD of the B.1.617.1 group and the PYD1/EBY200 (Empty vector) group. Asterisks represent significance: **p < 0.01; *n* = 6, ns, no significant; ***: p< 0.001.

To assess T-cell responses, splenocytes were harvested from mice on day 120, 5 weeks after the fifth immunization with B.1.617.1 mRBD, and stimulated with wild-type RBD protein. As shown in **Figures 5A** **, B**, after stimulation with RBD antigen, more IFN-γ-secreting cells were observed in the B.1.617.1 mRBD group than the empty vector control group. Intracellular cytokine staining showed that the frequency of cytokine-secreting CD4^+^ T cells in the spleen after RBD restimulation was much higher in the B.1.617.1 mRBD group than the empty vector control group (**Figure 5C**). However, the increase in the frequency of cytokine-secreting CD8^+^ T cells was not significantly different between the two groups (**Figure 5C**). The total level of TNF-α secreted by CD4^+^ T cells was significantly increased in mice inoculated with the B.1.617.1 mRBD vaccine compared with the empty vector group (**Figure 5D**). The frequency of CD4^+^ T cells simultaneously producing two or three cytokines was elevated in the spleens of mice immunized with the B.1.617.1 mRBD. A more detailed analysis revealed increases in the TNF-α^+^IL-2^+^IFN-γ^–^ T cell subset and a new TNF-α^+^IL-2^–^IFN-γ^+^ subset in the B.1.617.1 mRBD group. In addition, the B.1.617.1 mRBD group showed a lower proportion of the monofunctional IL-2^+^ subset compared with the empty vector group after stimulation with wild-type RBD *in vitro* (**Figure 5E**).

### Ethanol-treated *S*. *cerevisiae* surface-displayed wild-type RBD elicits humoral immune and specific T-cell responses with alternative inactivation treatment

**Figure 6:**
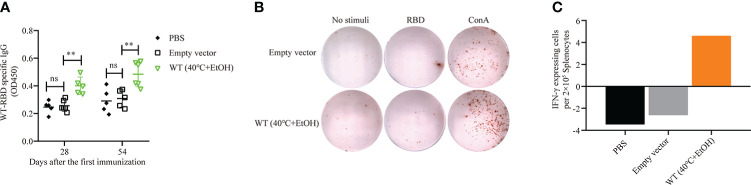
Humoral and cellular immune responses of pYD1-RBD (wild-type)/EBY200 recombinant yeast cells inactivated with 25% ethanol at 40°C. Female BALB/c mice were subcutaneously immunized with recombinant yeast or PBS on days 1, 15, and 31. **(A)** Plasma samples collected on days 28 and 54 after initial immunization were used to determine the levels of RBD-specific IgG by ELISA. Data points represent individual animals. **(B)** Blood samples were collected on day 54 after initial immunization, and samples from the same group were pooled. Aliquots of 2 × 10^5^ PBMCs isolated from each pooled blood sample were stimulated with SARS-CoV-2 S-RBD antigen, concanavalin A (Con A), or medium only. IFN-γ-expressing cells were counted. Each dot represents a single cell that secretes IFN-γ. **(C)** Quantification of the number of IFN-γ-expressing cells per 2 × 10^5^ PBMCs. PBMC: peripheral blood mononuclear cell. For statistical analysis, *t*-test was performed to compare empty vector and PBS groups. Asterisks represent significance: **p < 0.01; *n* = 6, ns, no significant.

The results outlined above showed that of four recombinant yeasts expressing wild-type RBD and mRBDs, only the B.1.617.1 mRBD successfully elicited both humoral and cellular immune responses. This may have been due to the inconsistent expression of antigen RBD or heat treatment at 60°C, which may damage the yeast cells. Therefore, we examined the effects of an alternative inactivation method with ethanol in wild-type RBD recombinant yeast. For inactivation, pYD1-RBD/EBY200 cells were incubated with 25% or 35% ethanol at 40°C for 1 h. On immunofluorescence analysis, yeast cells treated with 35% ethanol at 40°C for 1 h had no fluorescent signal, suggesting that the RBD may have detached from the yeast cell surface (**Figure S1B**). However, treatment with 25% ethanol at 40°C did not affect the anchoring of RBD to the surface of yeast cells (**Figure S1C**). Then, we examined the effects of 25% ethanol treatment on the growth of yeast cells, and confirmed that it completely inactivated yeast cells to the same extent as heat treatment at 60°C (**Figure S2**). The group vaccinated with pYD1-RBD/EBY200 recombinant yeast cells inactivated by 25% ethanol produced a significantly higher RBD-specific IgG antibody titer on days 28 and 54 than controls (*p* < 0.01) (**Figure 6A**). As shown in **Figures 6B** **, C**, after stimulation with RBD peptide, more IFN-γ-secreting cells were observed in the groups vaccinated with pYD1-RBD/EBY200 recombinant yeast cells inactivated by 25% ethanol than in the two control groups (PBS or pYD1/EBY200). These observations indicated that yeast vaccines with surface-displayed RBD can induce a cellular immune response. Compared with the conventional inactivation by heat treatment at 60°C, wild-type RBD recombinant yeast vaccine failed to successfully stimulate humoral and cellular immune responses, but ethanol-inactivated recombinant yeast successfully stimulated humoral and cellular immune responses. Therefore, this method represents an effective alternative means of inactivating yeast cell vaccines.

### Effects of glycosylation on *S*. *cerevisiae* cell surface-displayed RBD

**Figure 7:**
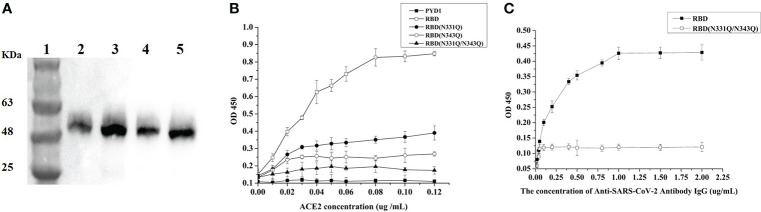
Glycosylation analysis of N331 and N343 of RBD. **(A)** Western blotting analysis of RBD protein and its mutant variants expressed on the surface of *S*. *cerevisiae* EBY200; lane 1: Mark, lane 2: PYD1-RBD/EBY200, lane 3: PYD1-RBD (N331Q)/EBY200, lane 4: PYD1-RBD (N343Q)/EBY200, lane 5: PYD1-RBD (N331Q&N343Q)/EBY200. **(B)** Binding of SARS-CoV-2 Spike protein RBD and its deglycosylation mutant variants to human ACE2. **(C)** Neutralizing activity of RBD and RBD (N331Q/N343Q) with SARS-CoV-2 IgG.

SARS-CoV-2 utilizes a glycosylated Spike (S) protein to bind to ACE2 to mediate host cell entry (30). The RBD of the S protein was shown to be the immunodominant antigen of SARS-CoV-2 and to efficiently bind to ACE2 (31). NMR experiments confirmed that S protein has 22 N-linked glycosylation sites, including N331 and N343 in the RBD region (32, 33). To determine the effects of glycosylation of N331 and N343 of RBD in *S*. *cerevisiae* EBY200 on binding affinity to ACE2, we replaced N331 and N343 with N331Q and N343Q through site-directed mutagenesis. The expression of RBD and its mutants was analyzed by Western blotting (**Figure 7**). As shown in **Figure 7A**, when we replaced N331 with N331Q (lane 3) or N343 with N343Q (lane 4), the molecular weight was slightly decreased compared with RBD (lane 2), suggesting less glycosylation in the mutated RDBs. When both N331 and N343 were replaced simultaneously with N331Q and N343Q (lane 5), the molecular weight showed a further decrease indicating that RBD indeed underwent glycosylation in yeast. As the glycosylation of RBD protein may cause a conformational change and thus affect binding to ACE2, we examined the binding affinities of different RBD mutants to ACE2. As expected, the wild-type RBD showed strong binding affinity to ACE2 (**Figure 7B**). However, a single deglycosylation at each of the N-linked glycosylation sites caused a significant decrease in binding affinity to ACE2 by 2.7-fold in the N331Q mutant and by 4-fold in the N343Q mutant. Double deglycosylation in RBD mutant N331Q/N343Q resulted in a marked reduction of binding affinity to ACE2 (**Figure 7B**). In addition, we evaluated the neutralizing activity of the deglycosylated RBD (N331Q and N343Q) with SARS-CoV-2 IgG. The results of ELISA demonstrated that deglycosylation of RBD reduced the neutralizing activity of IgG by about 20-fold, allowing escape from neutralizing antibodies (**Figure 7C**). These results indicate that the modification of glycosylation at N331 and N343 in the RBD region of the S protein plays a vital role in the binding of SARS-CoV-2 to ACE2 and thus in host cell entry. In addition, these observations highlight that the choice of expression host should be considered carefully when the antigen protein has glycosylation sites.

## Discussion

With ongoing cycles of transmission around the world, SARS-CoV-2 variants have arisen with mutations, including the Spike protein gene, which is the primary antigenic target of all SARS-CoV-2 vaccines currently in use (34). From the Alpha and Delta variants to the current Omicron variant (35), SARS-Cov-2 is mutating much faster than vaccine development. Therefore, the development of a platform for rapid vaccine production in response to the continuous mutations of SARS-CoV-2 is necessary. In addition, while more than 9 billion doses of vaccines have been administered globally, the distribution of vaccines is severely skewed. Half of the planet is currently unvaccinated, and only 4% of the population in low-income countries has been vaccinated (36). Reducing the cost of vaccine preparation is an important way to break the inequitable distribution of vaccination in low-income countries. Due to its rapid growth, low cost, and non-pathogenicity, yeast has been engineered to express various viral antigens as potential vaccines (37) and has already achieved impressive results in vaccine applications against hepatitis B virus (38), influenza virus (39), and encephalitis virus (40). In this study, the RBD gene and its variants were chosen for display on the *S. cerevisiae* surface. The B.1.617.1 mRBD vaccine induced high levels of RBD-specific IgG antibodies, and had cross-reactivity against wild-type RBD as well as mRBDs of B.1.1.7 and B.1.351.1 variants (**Figures 4A** **, B**). However, the surface display of wild-type RBD and mRBDs of B.1.351.1 and B.1.1.7 did not successfully elicit immune responses in mice. This could be explained as the low immunogenicity of protein subunit vaccines. Although subunit vaccines have the advantages of high safety and no obvious side effects compared with other vaccine types, such as inactivated virus or viral vector vaccines, they tend to induce lower levels of immunogenicity. Dai et al. demonstrated that coronavirus RBD dimer immunogen elicits stronger immunogenicity than the monomer in rodents (41). It is necessary to enhance the immunogenicity through artificial optimization of RBD in future studies.

While our manuscript was in preparation, Gao et al. (42) developed a yeast surface-based RBD vaccine that can produce significant humoral and mucosal reactions in mice by oral administration. However, as their system used the *gal* promoter in *S*. *cerevisiae*, it is necessary to replace the glucose carbon source with galactose in the later stages of culture, which greatly increases the cost and causes the production cycle to exceed 72 h. To save costs and facilitate rapid production, we knocked out *gal80* of *S*. *cerevisiae* EBY100 so the gene expression could be induced directly in glucose medium without adding galactose and successfully shortened the conventional 72-h culture period to 24 h in this study. Moreover, taking usage of glucose as a carbon source not only shortens the culture period but also allows for high-density culture (43), which would greatly reduce the cost. In addition, as heat treatment at 60°C may damage yeast cells, we examined an alternative inactivation treatment using ethanol. Our results indicated that the ethanol-inactivated wild-type RBD successfully stimulated humoral and cellular immune responses in mice (**Figure 6**). Unlike the traditional 60°C heat treatment inactivation, this alternative method of inactivating yeast cells with ethanol seemed to be more effective compared with heat treatment (**Figure 6**).

The storage and transportation of vaccines have always been challenging, and are commonly referred to as the “cold chain” problem, in which cold storage conditions (usually 2°–8°C) should be maintained at each step. Maintaining a continuous cold chain may bring the cost increase of vaccines by about 80% (44). For example, the COVID-19 mRNA vaccine developed by Moderna/NIAID should be stored at –20°C (45), while Pfizer and BioNTech mRNA vaccines require storage at –70°C during the entire distribution process from the manufacturer’s stores to its intended destination (7). To save storage costs, we prepared recombinant *S*. *cerevisiae* pYD1-RBD/EBY200 as a lyophilized yeast powder, which is a simple and effective method of long-term preservation of vaccines under non-refrigerated conditions (18). ELISA quantification showed that storage at room temperature for 1 month had almost no effect on the protein stability of lyophilized yeast powder (**Figure 3C**). After 10 months of storage at 4°C, the RBD-specific fluorescence signal of the lyophilized yeast powder could still be detected (**Figures 3D** **, E**). Overall, we have developed a simple and effective method for long-term storage of immunogens without freezing based on yeast surface display.

Prevention of infection is related to the induction of functional specific antibodies, especially antibodies neutralizing the RBD : ACE2 interaction. However, the T-cell response also plays an important role in COVID-19 mitigation, especially in the patients without measurable humoral responses (Chang-Monteagudo et al., 2021). In our study, SARS-CoV-2-specific CD4+ T-cell responses were more dominant than CD8+ T-cell responses after vaccination mice using yeast with the surface-displayed B.1.617.1 mRBD (**Figure 5C**). Actually, studies on adaptive immune responses to SARS-CoV-2 demonstrated that CD4+ T cells had a strong response to Spike protein and were correlated with the titers of the anti-SARS-CoV-2 antibody, and it is reported that the SARS-CoV-2-specific CD4+ T cells are detectable in 100% of recovered COVID-19 patients, while the detection rate of CD8+ T cells is only about 70% (46). Additionally, CD4+ T-cell responses associated with milder disease in acute and convalescent COVID-19 patients (47), showing its role in controlling and resolving a primary SARS-CoV-2 infection.

T cells that simultaneously produce two or more cytokines are defined as multifunctional T cells (48). SARS-CoV-2-specific CD4+ Th1 cells that produce IFNγ, TNF-ɑ, and/or IL-2 can be detected in vaccinators or convalescents (46, 47, 49), which is similar to other viral infections. Multifunctional Th1 cells play an essential role in the anti-SARS-CoV-2 response. We also found that vaccination with B.1.617.1 mRBD could induce an increase in the frequency of multifunctional T cells, including TNF-α+IL-2+IFN-γ– and TNF-α+IL-2-IFN-γ+ (**Figure 5E**), in addition to inducing the increase of TNF-α+ CD4+ T cells (**Figure 5D**). In a clinical study of 109 outpatients with COVID-19, the magnitude and quality of SARS-CoV-2-specific CD4+ T-cell response showed a shift, from IFNγ-producing cells to TNF-α-producing cells over time. The TNF-α^+^ CD4^+^ T cells dominated the SARS-CoV-2 specific T-cell response at later time points in patients, and were associated with persistent antibodies (50). This reminds us that IFN-γ+ CD4+ T cells may be detected at an early time point rather than at the immunological endpoint (D120) (**Figure 5D**). In addition to Th1 cells, circulating Tfh (cTfh) cells have been found in patients infected with acute SARS-CoV-2 (51, 52); however, their relationship with antibody response remains unclear. This suggests that we need to improve the CD4+ T-cell response and further study the CD4+ T-cell subsets to clarify which subsets are highly correlated with the maintenance of antibodies after immunization or infection, and provide direction for the improvement and optimization of vaccines.

In summary, we have constructed a yeast display platform for low-cost and rapid manufacture of SARS-CoV-2 S-RBD and variant vaccines. The candidate *S*. *cerevisiae* pYD1-B.1.617.1/EBY200 vaccine successfully elicited humoral and cellular immune responses. This method has the potential to solve the global problems of vaccine supply and large-scale production.

## Data availability statement

The original contributions presented in the study are included in the article/**Supplementary Material**. Further inquiries can be directed to the corresponding author/s.

## Ethics statement

This study was reviewed and approved by The Animal Ethics Committee of the School of Basic Medical Sciences, Fudan University (approval No. FE21113).

## Author contributions

ZZ, GZ, XY, and HZ initiated and coordinated the project. HX completed plasmid construction, strain culture, protein expression, biochemical analysis, and vaccine preparation. LZ and HZ completed animal immunity experiment and data analysis. HX and LZ wrote the manuscript with contributions from all authors. PW, XY, YY, and ZZ completed the revision of the manuscript. All authors contributed to the article and approved the submitted version.

## Funding

This work was financially supported by the National Key Research and Development Program of China (Grant No. 2018YFA0900700), the National Natural Science Foundation of China (No. 32071425), the Strategic Priority Research Program of the Chinese Academy of Sciences (Grant No. XDB27020206), the Strategic Biological Resources Service Network Plan of the Chinese Academy of Sciences (Grant No. KFJ-BRP-009; KFJ-BRP-017-60), and the Shanghai Municipal Science and Technology Major Project on Infectious Diseases.

## Acknowledgments

We thank Prof. Sibao Wang (CAS Center for Excellence in Molecular Plant Sciences, Chinese Academy of Sciences) for equipment support in immunofluorescence microscopy. We thank Dr. YingYing Chen (Shanghai Institute of Immunology, Department of Microbiology and Immunology, Shanghai Jiao Tong University School of Medicine) and Dr. XiaoYong Fan (Shanghai Public Health Clinical Center, Fudan University) for insightful suggestions on techniques and design of immunization experiments. We thank Qinglian Xie (Shanghai Institute of Nutrition and Health, Chinese Academy of Sciences), Xiaoting Zhu (Zhejiang Hongguan Bio-pharma Co., Ltd.), and Chenchen Feng (Yueyang Hospital of Integrated Traditional Chinese and Western Medicine, Shanghai University of Traditional Chinese Medicine) for technical assistance.

## Conflict of interest

Author LZ was employed by Zhejiang Hongguan Bio-pharma Co., Ltd.

The remaining authors declare that the research was conducted in the absence of any commercial or financial relationships that could be construed as a potential conflict of interest.

## Publisher’s note

All claims expressed in this article are solely those of the authors and do not necessarily represent those of their affiliated organizations, or those of the publisher, the editors and the reviewers. Any product that may be evaluated in this article, or claim that may be made by its manufacturer, is not guaranteed or endorsed by the publisher.
