# Supplementary material for: Display of receptor-binding domain of SARS-CoV-2 Spike protein variants on the Saccharomyces cerevisiae cell surface
Source: Front Immunol. 2022 Aug 12;13:935573. doi: 10.3389/fimmu.2022.935573 (PMC9412237; doi:10.3389/fimmu.2022.935573)
Supplement: Supplementary file 1 [file DataSheet_1.docx]

**Display of receptor-binding domain of** **SARS-CoV-2 Spike protein and its variants on the *Saccharomyces cerevisiae* cell surface**

Hongguan Xing ^1,2†^, LiYan Zhu^3,4†^, Pingping Wang^2,^ Guoping Zhao^2,3^, Zhihua Zhou^2^, Yi Yang ^1^,

Hong Zou^5*^ , Xing Yan^1,2*^

^1^ Shanghai Key Laboratory of Chemical Biology, School of Pharmacy, East China University of Science and Technology, Meilong Rd 130, Shanghai, 200237, China

^2^ Chinese Academy of Sciences -Key Laboratory of Synthetic Biology, Chinese Academy of Sciences Center for Excellence in Molecular Plant Sciences, Institute of Plant Physiology and Ecology, Chinese Academy of Sciences, Shanghai 200032, China

^3^ Key Laboratory of Medical Molecular Virology of the Ministry of Education/National Health
Commission, School of Basic Medical Sciences, Shanghai Medical College, Fudan University,Shanghai, China

^4^ Zhejiang Hongguan Bio-pharma Co., Ltd., Department of Process Technology, Jiaxing 314500, China.

^5^ Chinese Academy of Sciences Engineering Laboratory for Nutrition, Shanghai Institute of Nutrition and Health, Chinese Academy of Sciences, Shanghai, China

Running title: Display of mRBDs on yeast surface

^†^ Hongguan Xing, Liyan Zhu contributed equally to this work and share first authorship

^*^Correspondence: [Hong](mailto:Hong) Zou, Email: hzhou01@sibs.ac.cn; Xing Yan, Email: yanxing@cemps.ac.cn


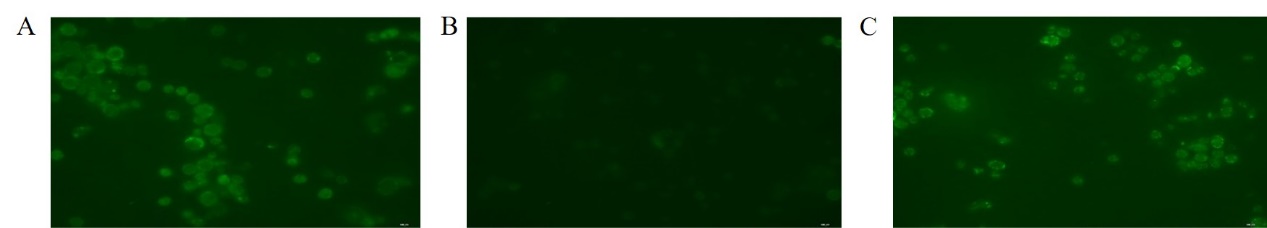


Fig. S1 The effects of different treatment conditions on the stability of Aga2-RBD fusion protein on yeast surface; A: Untreated; B: 35% ethanol, incubate at 40℃ for 1 h; C: 25% ethanol, incubate at 40℃ for 1 h.


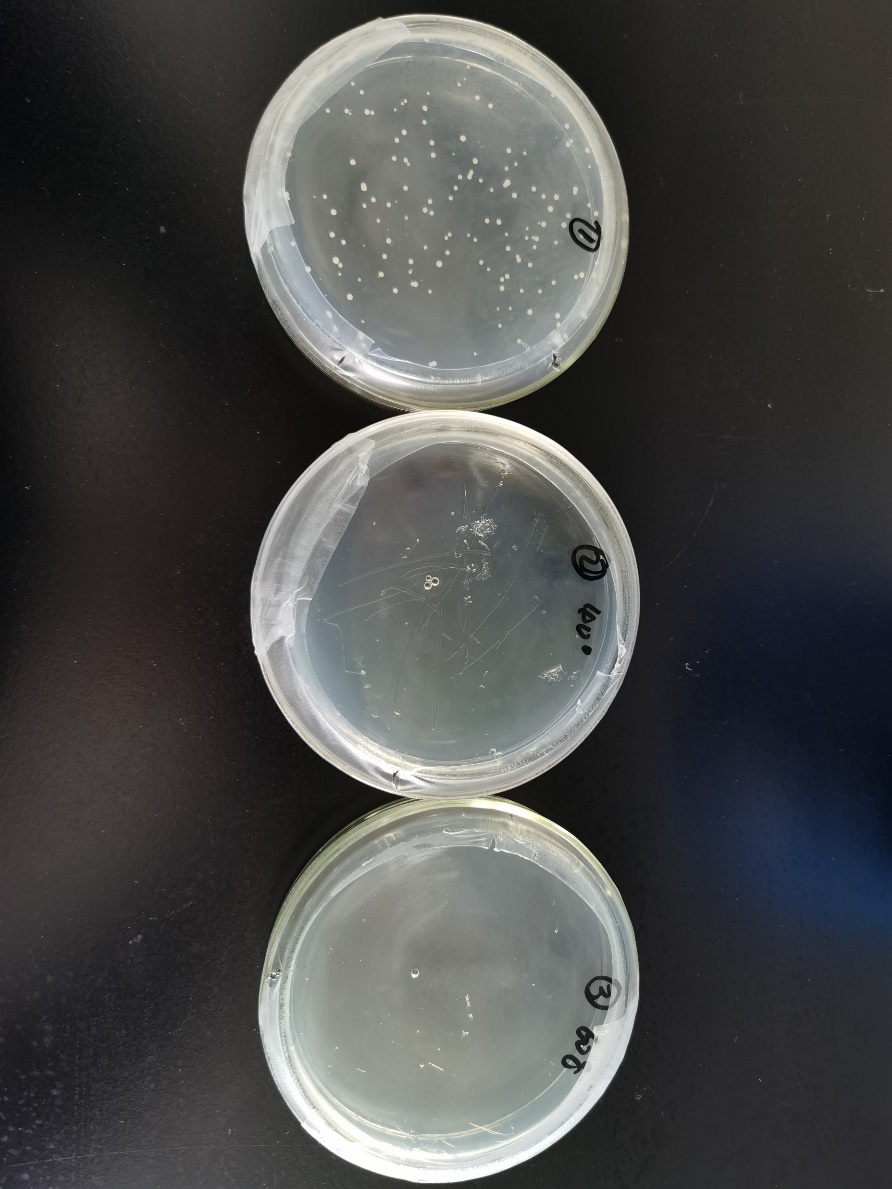


Fig. S2 The effect of different treatment conditions on *S. cerevisiae* EBY 200 cells；①：Untreated; ②: 25% ethanol, incubate at 40℃ for 1 h; ③: incubate at 60℃ for 1 h.
